# Mathematical Modelling of Thermal Process to Aquatic Environment with Different Hydrometeorological Conditions

**DOI:** 10.1155/2014/678095

**Published:** 2014-06-02

**Authors:** Alibek Issakhov

**Affiliations:** Al-Farabi Kazakh National University, Almaty 050040, Kazakhstan

## Abstract

This paper presents the mathematical model of the thermal process from thermal power plant to aquatic environment of the reservoir-cooler, which is located in the Pavlodar region, 17 Km to the north-east of Ekibastuz town. The thermal process in reservoir-cooler with different hydrometeorological conditions is considered, which is solved by three-dimensional Navier-Stokes equations and temperature equation for an incompressible flow in a stratified medium. A numerical method based on the projection method, divides the problem into three stages. At the first stage, it is assumed that the transfer of momentum occurs only by convection and diffusion. Intermediate velocity field is solved by fractional steps method. At the second stage, three-dimensional Poisson equation is solved by the Fourier method in combination with tridiagonal matrix method (Thomas algorithm). Finally, at the third stage, it is expected that the transfer is only due to the pressure gradient. Numerical method determines the basic laws of the hydrothermal processes that qualitatively and quantitatively are approximated depending on different hydrometeorological conditions.

## 1. Introduction

Many years in the study of hydrodynamics of lakes and reservoirs were two independent directions; one of them is the analysis of data and the other is mathematical modelling. Natural experiments—observations, although made in a variety of complex conditions, were passive, as they do not enable you to manage the experiment; they do not seem possible based on their prediction of hydrophysical processes. Performed experiments and calculations were not convincing enough, because the models were not calibrated and are usually not verified. Most often, operated models are available to the developer or the user but are not optimal from the point of view of the problem.

The lakes are observed not only by the wave movement but also by the vortices which have spatial scales that could be compared to the size of the reservoir, or to the portions thereof, mushroom formations, meanders, jet currents, trails, and torches waters of different origins. As shown by observations of flows and a number of indirect factors, there is significant variability of dynamic processes typical for areas with complex geometry of bottom and coasts. These topographic features are manifested in the formation of eddies and meandering streams. Spectrum observation of lakes' vortices and wave motions widens and extends from microvortices leading to energy dissipation to large-scale eddies, covering the entire lake. Description of large-scale topographic eddies could be done by using the conservation law of potential vortices.

One of the most effective methods of studying the hydrodynamics of the lake is a method of mathematical modeling. In some cases, this may be the only tool to predict changes in the hydrological regime and lake ecosystems, for example, when studying the changes that may occur in spatial redistribution of water, while constructing waterworks and other events associated with the use of water objects.

Mathematical models can be classified according to several criteria. It can be classified according to their model “dimension”: one-dimensional (vertical or horizontal), two-dimensional (horizontal or vertical plane), and three-dimensional model.

The most simple are one-dimensional models that are commonly used for modeling of currents in rivers. Two-dimensional models are used to study wind and seiche flows, storm surges, and so forth.

Lick [1] proposes to consider the following types of mathematical models of wind currents like (1) integrated model (full flow), in which vertical integration over the flow is accounted but vertical profile of flows is not modeled, (2) stationary models of wind currents for constant and variable density of water, and (3) nonstationary model for barotropic and baroclinic lakes.

To study the wind currents, Shang et al. [2] divided the models to Ekman, integrated by vertical direction, multilevel, and multilayered. In addition to these models, the dynamic method and a variety of three-dimensional thermohydrodynamic models are used for calculation of lakes' flows.

It is accepted to divide modelling into two classes—prognostic and diagnostic. In the first class, the formation of interconnected flow fields, temperature, and boundary layers of the atmosphere and the lake are simulated. Solution of this problems class involves great difficulties of a numerical simulation of unsteady nonlinear partial differential equations. The use of data obtained from the observation of temperature and wind fields greatly simplifies the problems of circulation in lakes. This is the meaning of diagnostic solutions, which are widely used in the class of oceanographic problems and subsequently for the study of flows in deep stratified lakes.

It can be attributed to the special class models that simulate mesoscale movements such as inertial oscillations, seiche flows, Kelvin, topographic and Poincare waves, the flow in the zone of coastal upwelling, and coherent structure.

The model originally developed for the ocean is widely used in the modeling of the dynamics of lake, especially large and deep. At the same time, taking into account the specific features of the thermodynamics of lakes and their small size compared to the ocean, the presence of the maximum density at about 4°C is remarkable property of the largest stratified lakes that leads to the formation of the thermal bar, which mostly determines the dynamics of the lake as well as its chemical and biological mode. Moreover, it is important that the low tide, the beta effect, and absence of Rossby waves that are typical for the ocean could be related to the small size lakes too. However, the heterogeneity of the bottom topography in the lakes contributes to the formation of topographic Rossby waves, analogues oceanic Rossby waves. In closed basins, standing waves, seiche, which, if sufficiently large, lakes split into fashion with the formation of waves Kelvin and Poincare, play an important role.

## 2. Background

Getting electricity from thermal power plants (TPP) had a higher priority than its impact on the environment. Technology of production of electrical energy from power plant is connected with a lot of waste heat released into the environment. Today, the problem of influence on the nature by power is particularly acute because the pollution of the atmosphere and hydrosphere increases each year.

The energy consumption scale is increasing year by year; as a result, negative impact of energy on the environment also increases. Before, to get energy, primarily guided feasibility was economic costs, but now, in the construction and operation of energy, the most important issue is their impact on the environment.

Another problem of TPP is thermal pollution to the reservoir or lake. Dropping hot water-push chain reaction that begins reservoir overgrown with algae, it violates the oxygen balance, which in turn is a threat to the life of all its inhabitants. Thermal power plants with cooling water shed 4–7 kJ of heat for 1 kW/h electricity generation. Meanwhile, according to the Health Standards, discharged warm water from TPP should not rise higher than 3°C in the summer and 5°C in the winter from the initial temperature of the reservoir.

Spread of harmful emissions from TPP depends on several factors: the terrain, environmental temperature, wind speed, cloud cover, precipitation intensity, and so forth. Existing meteorology conditions like wind velocity and so forth increase the thermal pollution area.

Large proportion of electricity (81.3%) in the world is produced by thermal power plants. Therefore, emissions of this type of power plants to the atmosphere and hydrosphere provide the greatest amount of anthropogenic contaminants in it.

Thermal pollution of reservoirs or lakes water that cause multiple violations of their state is one representation of environment danger. Thermal power plants generate energy through turbines, driven by hot steam, while the exhaust steam is cooled by water. Therefore, from the power plants in the reservoirs or lakes, this process is continuously transferred from the water flow temperature at 8–12°C above the temperature of the water in the reservoir. Large TPP sheds till 90 m³/s of heated water. For example, according to estimates of German and Swiss scientists, the possibility of rivers of Switzerland and the upper flows of the Rhine on the heating have been exhausted. Hot water at any place of the river should not exceed more than 3°C maximum temperature of the river water, which is assumed to be 28°C. Following these conditions, the power station of Germany, constructed on the Rhine, Inna, Weser, and Elbe, is limited by 35 000 MW. Thermal pollution can lead to tragic consequences. Scientists predict change in the characteristics of the environment in the next 100–200 years.

Let us consider hydrosphere pollution. Heat from TPP mainly is given to the environment from the water-cooled condenser steam turbines. The value of heat released to the environment depends on the capacity of thermal power plants. If we consider power plants, from 40 to 70% of the amount of diverted energy to the environment is taken from thermal energy released by combustion fuel. Cooling water in and direct-flow-back scheme of intake and discharge of water are limited by the local allowable increase in the temperature of the source water (river, lake, or reservoir) in the thermal effects. Water supply system has a number of features of TPP. Almost all of the water up to 95% of total cost is applied to cool the condenser coils and auxiliary steam turbines. With up to 5% of the total value of the water supply to the thermal power plant equipment is generally irreversible consumption. As a rule, the main building of the condensing power plant is located directly at the shore line of the river-, lake-, or reservoir-cooler. Water is supplied to the main unit of heat removal to the environment pumping stations. After heating it in condensers and heat exchangers, water is discharged to the surface of the water. However, this amount of water is heated.

## 3. Study Area

In this work, Ekibastuz SDPP-1 is considered, which is located in Pavlodar region, 17 km to the north-east of the Ekibastuz city, Kazakhstan. It is taken as an example of the impact of TPP on the reservoir-cooling. Technical water supply of SDPP-I was carried on the recircuit with cooling water circulation. The surface of the reservoir is at 158.5 m above the sea, the area is 19.6 km^2^, the maximum size is 4 × 6 km, the average depth is 4.6 m, maximum depth is 8.5 m at the intake, and the volume of the reservoir is 80 million m^3^. Moreover, combined type of selective intake and discharge is used in the body of the water. Discharged water enters the prechannel mixer and then through a filtration dam uniformly enters the reservoir-cooling. Water intake is at a distance of 40 m from the dam and the depth is 5 m. Design flow of water is 120 m^3^/s, and the actual flow rate varies depending on the mode of TPP within 80–120 m^3^/s.

## 4. Materials and Methods

Now hydrophysical problems associated with discharge of heated water into reservoirs in the operation of thermal or nuclear power plant become important. The discharged heated water is an important hydrologic and environmental problem. That is the reason why we have to predict and control the temperature of the water and the content of impurities in the reservoirs and rivers. Successful study of the processes occurring in the reservoir involves a complex study of the problem: instead of measurements taken from hydrothermal parameters, it is better to use mathematical modeling of the processes and then compare results of the modelling of physical process in laboratory and natural conditions. To construct mathematical models, it is necessary to consider the main characteristic of the flow in a reservoir-turbulent fluid motion. This in turn merges with one of the major problems of hydrodynamics-theory of turbulence.

Thermal and nuclear power plants both require reservoirs. Electricity production is increasing worldwide and especially from postwar period, doubling every 7–10 years. Large amounts of water are required to operate these plants for cooling units, in average 35–40 m^3^/s for 1 million kW of installed capacity. Hence, it is evident that for the thermal power plants of 2.4 million kW 70–160 m^3^/s of water is required. Therefore, water supply becomes important when we choose to build coal and nuclear power plants. Naturally, the large thermal power plants should be located on the banks of large rivers, ponds, lakes, or artificial reservoirs. The creation of artificial reservoirs requires large investment, so power stations tend to have existing reservoirs and lakes. Often, industrial facilities located on the shores of lakes and reservoirs disposed of with warm water waste products in the form of impurities. If we consider that in the most developed countries for 2010–2020 years cooling thermal power plants and industrial facilities will use more than 10% of water resources, the problems of optimal and efficient use of water reservoir for cooling are in great importance. In solving these problems, there is a need to be able to predict and control the temperature of the water and the spread of passive pollutants of reservoir.

In recent years, association put tough restrictions to protection of the environment. The designer of artificial reservoir usually has to follow the rules, which limit the size of “zone transfer” by fault of hot water so that it does not exceed half the width of the river and occupy no more than half of the total cross-sectional area and flow. If these rules are not followed, it may lead to short-term or long-term stop of power plant. That is why the accuracy requirements to constructive analysis are very strict. In fact, emerging with the hydrodynamic problem, the process can be described as fully three-dimensional, with irregular boundaries, with the presence of buoyancy and the velocity of the main flow, which can vary by an order, sometimes so fast that the important role plays the effects of nonstationary. In addition, there are large areas of recycling if certain combinations of conditions were applied when the fault-heated water is almost drawn into the upstream region of cooling water. The result could be a significant loss of total operating efficiency of the system.

## 5. Mathematical Model

From the above, it follows that the construction of a mathematical model relevant to real processes in the reservoir-cooler is quite a challenge.

There are many mathematical and numerical models that have been developed to simulate distribution temperature after launching TPP in reservoir-cooler [3–5].

The impact of thermal or nuclear power plant on the hydrological and biological conditions of the reservoir is various. Most of all, thermal pollution often reaches 30–35 degrees of heated water. This increases the water temperature and adversely affects hydrobiological condition, self-purification of water quality of the reservoir. In the reservoir-cooler, spatial change of temperature is small. Therefore, stratified flow in the reservoir-cooler can be described by equations in the Boussinesq approximation. For the mathematical modeling of the system motion, continuity and temperature equations are considered. The development of spatial turbulent stratified flows in reservoir-cooler is considered in [6–9]. Three-dimensional model is used for mathematical modelling of temperature distribution in the reservoir-cooler [10–12]. Consider

(1) $$\begin{array}{c} \frac{\partial u_{i}}{\partial t}+\frac{\partial u_{j}u_{i}}{\partial x_{j}}=-\frac{\partial p}{\partial x_{i}}+\nu \frac{\partial }{\partial x_{j}}\left(\frac{\partial u_{i}}{\partial x_{j}}\right)-\frac{\partial \tau_{ij}}{\partial x_{j}}+\beta g_{i}\left(T-T_{0}\right), \end{array}$$

(2) $$\begin{array}{c} \frac{\partial u_{j}}{\partial x_{j}}=0, \end{array}$$

(3) $$\begin{array}{c} \frac{\partial T}{\partial t}+\frac{\partial u_{j}T}{\partial x_{j}}=\frac{\partial }{\partial x_{j}}\left(\chi \frac{\partial T}{\partial x_{j}}\right), \end{array}$$

(4) $$\begin{array}{c} \text{where}\;\tau_{ij}=\bar{u_{i}u_{j}}-\bar{u_{i}}\;\bar{u_{j}}, \end{array}$$

where *g*_*i*_ is the gravity acceleration, *β* the coefficient of volume expansion, *u*_*i*_ velocity components, *χ* thermal diffusivity coefficient, *T*_0_ the equilibrium temperature, and *T* deviation of temperature from the balance.

This system of equations was filtered by using large eddy simulation (LES) method. The basic idea of LES method is a mathematical division of the large and small universal vortices. This procedure can be performed through spatial averaging, that is, to define the field of large-scale quantities by filter. Consider

(5) $$\begin{array}{c} \bar{u}\left(x,t\right)=\overset{}{\underset{V}{\int }}G\left(r,x\right)u\left(x-r,t\right)dr, \end{array}$$

where **u** = (*u*_1_, *u*_2_, *u*_3_)—vector of velocity components, sign “dash” denotes averaging, **x** = (*x*_1_, *x*_2_, *x*_3_)—vector of coordinate system, **r** = (*r*_1_, *r*_2_, *r*_3_)—vector of coordinate system by which the integration is done, *V* is volume of integration, and *G*(**r**, **x**) is filter function with characteristic length scale such that

(6) $$\begin{array}{c} \overset{}{\underset{V}{\int }}G\left(r,x\right)dr=1. \end{array}$$

Small-scale fluctuations are as follows:

(7) $$\begin{array}{c} u^{\prime }\left(x,t\right)=u\left(x,t\right)-\bar{u}\left(x,t\right), \end{array}$$

and, in many cases, depending on the filter function, it looks like

(8) $$\begin{array}{c} \bar{u^{\prime }}\left(x,t\right)\neq 0. \end{array}$$

There are different approaches to characterize the filter:

(i) “Box” filter:
  (9) $$\begin{array}{c} G\left(r\right)=\frac{1}{\Delta^{3}}\{\begin{array}{cc} 1, & \left|r\right|\leq \frac{\Delta }{2}\\ 0, & \left|r_{i}\right|>\frac{\Delta }{2}, \end{array}\;\forall i=1,2,3. \end{array}$$
(ii) Gaussian filter:
  (10) $$\begin{array}{c} G\left(r\right)=\overset{3}{\underset{i=1}{\prod }}{\left(\frac{6}{\pi \Delta^{2}}\right)}^{1/2}\exp\left(-\frac{6r_{i}^{2}}{\Delta^{2}}\right). \end{array}$$
(iii) Cut filter:
  (11) $$\begin{array}{c} G\left(r\right)=\overset{3}{\underset{i=1}{\prod }}\frac{\sin\left(\pi r_{i}/\Delta \right)}{\pi r_{i}}. \end{array}$$

Δ is characteristic length of the filter, which is the order of the mesh size. It is usually taken like [13]

(12) $$\begin{array}{c} \Delta ={\left(h_{1}h_{2}h_{3}\right)}^{1/3},\\ \Delta ={\left(h_{1}^{2}+h_{2}^{2}+h_{3}^{2}\right)}^{1/2},\\ \Delta =\min\left(h_{1},h_{2},h_{3}\right), \end{array}$$

where *h*_*i*_ is step size corresponding to axes of a Cartesian coordinate system.

We start with regular LES corresponding to a “bar-filter” of Δ*x* width and an operator associating with the function $\bar{f}(\bar{x},t)$. Then we define a second “test filter” tilde of large 2Δ*x* width associating with $\overset{\sim }{f}(\overset{-}{x},t)$. Let us first apply this filter product to the Navier-Stokes equation. The subgrid-scale tensor of the field $\overset{\sim }{\bar{u_{i}}}$ is obtained from (4) by replacing filter bar to double filter and tilde filter:

(13) $$\begin{array}{c} \tau_{ij}=\overset{\sim }{\bar{u_{i}}}\;\overset{\sim }{\bar{u_{j}}}-\overset{\sim }{\bar{u_{i}u_{j}}}, \end{array}$$

(14) $$\begin{array}{c} l_{ij}=\overset{\sim }{\bar{u_{i}}}\;\overset{\sim }{\bar{u_{j}}}-\overset{\sim }{\bar{u_{i}}\;\bar{u_{i}}}. \end{array}$$

Now we apply the tilde filter to (4), which leads to

(15) $$\begin{array}{c} \overset{\sim }{\tau_{ij}}=\overset{\sim }{\bar{u_{i}}}\;\overset{\sim }{\bar{u_{j}}}-\overset{\sim }{\bar{u_{i}u_{j}}}. \end{array}$$

Adding (14) and (15) and using (13), we obtain

(16) $$\begin{array}{c} l_{ij}=\tau_{ij}-\overset{\sim }{\tau_{ij}}. \end{array}$$

We use Smagorinsky model expression for the subgrid stresses related to the bar filter and tilde-filter to get

(17) $$\begin{array}{c} \overset{\sim }{\tau_{ij}}-\frac{1}{3}\delta_{ij}\overset{\sim }{\tau_{kk}}=2C\overset{\sim }{A_{ij}},\;\text{where}\;\;A_{ij}={\left(\Delta x\right)}^{2}\left|\bar{S}\right|\bar{S_{ij}}. \end{array}$$

Further on, we have to determine *τ*_*ij*_, the stress resulting from the filter product. This is again obtained using Smagorinsky model, which yields to

(18) $$\begin{array}{c} \tau_{ij}-\frac{1}{3}\delta_{ij}\tau_{kk}=2CB_{ij},\;\text{where}\;\;B_{ij}={\left(2\Delta x\right)}^{2}\left|\overset{\sim }{\bar{S}}\right|\overset{\sim }{\bar{S_{ij}}}. \end{array}$$

Subtracting (17) from (18) and using Germano's identity, we get

(19) $$\begin{array}{c} l_{ij}-\frac{1}{3}\delta_{ij}l_{kk}=2CB_{ij}-2C\overset{\sim }{A_{ij}}, \end{array}$$

(20) $$\begin{array}{c} l_{ij}-\frac{1}{3}\delta_{ij}l_{kk}=2CM_{ij}, \end{array}$$

(21) $$\begin{array}{c} \text{where}\;\;M_{ij}=B_{ij}-\overset{\sim }{A_{ij}}. \end{array}$$

All the terms of (21) may now be determined by using $\bar{u}$. Unfortunately, there are five independent equations for only one variable *C* and thus the overdetermined problem. The first solution was proposed by Germano to multiply (21) tensorially by $\bar{S_{ij}}$ to get

(22) $$\begin{array}{c} C=\frac{1}{2}\frac{l_{ij}\bar{S_{ij}}}{M_{ij}\bar{S_{ij}}}. \end{array}$$

This provides finally dynamical evaluation of *C*, which can be used in the LES for the bar field $\bar{u}$ [13, 14].

Initial and boundary conditions are defined so that they satisfy the nonstationary three-dimensional equations of motion, continuity, and temperature.

## 6. Numerical Algorithm

Numerical solution of (1)-(2) is carried out on the staggered grid using the scheme against a stream of the second type. Moreover, compact approximation is used for convective terms [15, 16]. In view of the above with the proposed model of turbulence scheme of splitting on physical parameters is used to solve the problem. At the first stage, the transfer of momentum occurs only through convection and diffusion. Intermediate velocity field is solved by using fractional step method through the tridiagonal matrix method (Thomas algorithm) [16, 17]. The second stage is for pressure which is found by intermediate velocity field. Three-dimensional Poisson equation for pressure is solved by Fourier method in combination with the tridiagonal matrix method (Thomas algorithm) that is applied to determine the Fourier coefficients [16, 18]. At the third stage, it is supposed that the transfer is carried out only by the pressure gradient. The algorithm was parallelized on the high-performance system [16]. And we can mathematically propose this algorithm like

(23) (i) u→∗−u→nτ=−(∇u→nu→∗−νΔu→∗),(ii) Δp=∇u→∗τ,(iii) u→n+1−u→∗τ=−∇p.

For the first stage, intermediate velocity field is solved by using fractional step method through the tridiagonal matrix method (Thomas algorithm):

(24) fn+1/3−fnτ=12Λ1fn+1/3+12Λ1fn+Λ2fn+Λ3fn,fn+2/3−fn+1/3τ=12Λ2fn+2/3−12Λ2fn,f∗−fn+2/3τ=12Λ3f∗−12Λ3fn,

where the operators are like

(25) $$\begin{array}{c} \Lambda_{1}f=-\frac{\partial \left({\overset{-}{u}}_{1}^{\;n}f\right)}{\partial x_{1}}+\nu \frac{\partial^{2}f}{\partial x_{1}^{2}}-\frac{\partial \tau_{i1}}{\partial x_{1}},\\ \Lambda_{2}f=-\frac{\partial \left({\overset{-}{u}}_{2}^{\;n}f\right)}{\partial x_{2}}+\nu \frac{\partial^{2}f}{\partial x_{2}^{2}}-\frac{\partial \tau_{i2}}{\partial x_{2}},\\ \Lambda_{3}f=-\frac{\partial \left({\overset{-}{u}}_{3}^{\;n}f\right)}{\partial x_{3}}+\nu \frac{\partial^{2}f}{\partial x_{3}^{2}}-\frac{\partial \tau_{i3}}{\partial x_{3}},\\ f={\overset{-}{u}}_{i},\;i=1,2,3. \end{array}$$

The second stage is for pressure which is found by intermediate velocity field. Three-dimensional Poisson equation for pressure is solved by Fourier method for one coordinate in combination with the tridiagonal matrix method (Thomas algorithm). It is applied to determine the Fourier coefficients [16, 18]. The numerical algorithm for Poisson equation was parallelized on the high-performance system [16]. Mathematically, we can write Fourier method as follows:

(26) $$\begin{array}{c} p_{i,j,k}=\frac{2}{N_{3}}\overset{N_{3}}{\underset{l=0}{\sum }}\rho_{l}a_{i,j,l}\cos\frac{\pi kl}{N_{3}},\\ F_{i,j,k}=\frac{2}{N_{3}}\overset{N_{3}}{\underset{l=0}{\sum }}\rho_{l}b_{i,j,l}\cos\frac{\pi kl}{N_{3}}, \end{array}$$

where

(27) $$\begin{array}{c} a_{i,j,l}=\overset{N_{3}}{\underset{k=0}{\sum }}\rho_{k}p_{i,j,k}\cos\frac{\pi kl}{N_{3}},\\ b_{i,j,l}=\overset{N_{3}}{\underset{k=0}{\sum }}\rho_{k}F_{i,j,k}\cos\frac{\pi kl}{N_{3}}. \end{array}$$

Substituting the equation above into three-dimensional Poisson equation for pressure, we obtain the following expression:

(28) H2L122N3∑l=0N3ρl(ai+1,j,l−2ai,j,l+ai−1,j,l)Δx12cos⁡πklN3+H2L222N3 ×∑l=0N3ρl(ai,j+1,l−2ai,j,l+ai,j−1,l)Δx22 ×cos⁡⁡πklN3+2N3 ×∑l=0N3ρlai,j,lΔx32(cos⁡π(k+1)lN3−2cos⁡πklN3+cos⁡π(k−1)lN3)=2N3∑l=0N3ρlbi,j,lcos⁡πklN3.

Using the expression below,

(29) $$\begin{array}{c} \cos\frac{\pi \left(k+1\right)l}{N_{3}}+\cos\frac{\pi \left(k-1\right)l}{N_{3}}=2\cos\frac{\pi kl}{N_{3}}\cos\frac{\pi l}{N_{3}}, \end{array}$$

we can write (28) in the following form:

(30) H2L122N3∑l=0N3ρl(ai+1,j,l−2ai,j,l+ai−1,j,l)Δx12cos⁡πklN3 +H2L222N3∑l=0N3ρl(ai,j+1,l−2ai,j,l+ai,j−1,l)Δx22 ×cos⁡πklN3+2N3∑l=0N3ρlai,j,lΔx32(2cos⁡πlN3−2) ×2cos⁡πklN3=2N3∑l=0N3ρlbi,j,lcos⁡πklN3.

The last expression can be written at a fixed value *l* and divided by (2/*N*_3_)*ρ*_*l*_cos⁡(*πkl*/*N*_3_) and then we get

(31) H2L12ai+1,j−2ai,j+ai−1,jΔx12+H2L22ai,j+1−2ai,j+ai,j−1Δx22 +ai,jΔx32(2cos⁡πlN3−2)=bi,j.

Furthermore, this equation is transformed to the following form:

(32) −H2L22ai,j−1Δx22+[(H2L122Δx12+H2L222Δx22−1Δx22×(2cos⁡πlN3−2))ai,j−H2L12ai+1,j+ai−1,jΔx12]−H2L22ai,j+1Δx22=−bi,j.

In vector form, this equation can be written as follows:

(33) $$\begin{array}{c} -A_{j}{\vec{a}}_{j-1}+B_{j}{\vec{a}}_{j}-C_{j}{\vec{a}}_{j+1}={\vec{F}}_{j}, \end{array}$$

where *A*_*j*_, *B*_*j*_, *C*_*j*_ matrices and ${\vec{F}}_{j}$,  ${\vec{a}}_{j}$ vectors are taken in that form:

(34) a→j=|a0,j⋮aN1,j|,  Aj=|H2L221Δx220⋱0H2L221Δx22|,Bj=|H2L122Δx12+H2L222Δx22−1Δx22(2cos⁡πlN3−2)−H2L122Δx120−H2L121Δx12⋱−H2L121Δx120−H2L122Δx12H2L122Δx12+H2L222Δx22−1Δx22(2cos⁡πlN3−2)|,Cj=|H2L221Δx220⋱0H2L221Δx22|,  F→j=−|b0,j⋮bN1,j|.

Tridiagonal matrix algorithm (Thomas algorithm) for (33) looks like

(35) αj+1=(Cj−Ajαj)−1Bj,j=1,2,…N2−1, α1=C0−1B0,β→j+1=(Cj−Ajαj)−1(F→j+Ajβ→j),j=1,2,…N2, β→1=C0−1F→0,a→j=αj+1a→j+1+β→j+1,j=N2−1,…0, a→N2=β→N2+1.

The third is a correction stage; it is supposed that the transfer is carried out only by the pressure gradient. After calculating *a*_*i*,*j*,*k*_, pressure field values are found from (33). To calculate the sum (26), it is necessary to apply the fast Fourier transformation. That allows calculating the method by *O*(*N*ln⁡*N*). And finally the temperature equation (3) is also solved by using fractional step method through the tridiagonal matrix method (Thomas algorithm).

## 7. Results of Numerical Modelling

In the simulation, the mesh size of 200 × 200 × 200 was used.

**Figure 1:**
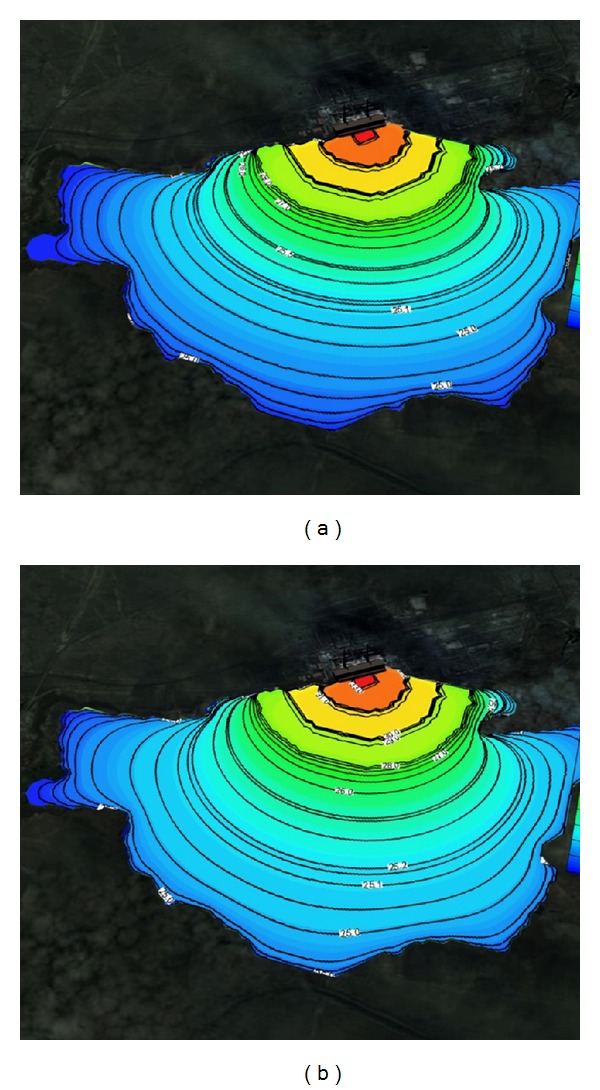
Outline and contours of temperature at 15 and 24 hours after launch of Ekibastuz SDPP-1 on the surface and the side view.

**Figure 2:**
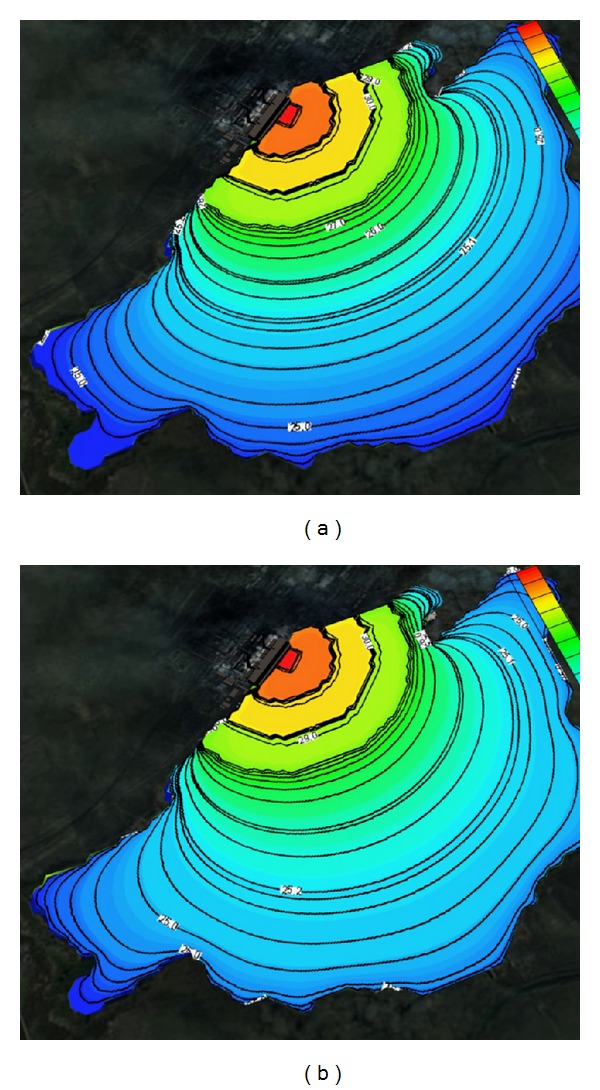
Outline and contours of temperature at 15 and 24 hours after launch of Ekibastuz SDPP-1 on the surface and the top view.

**Figure 3:**
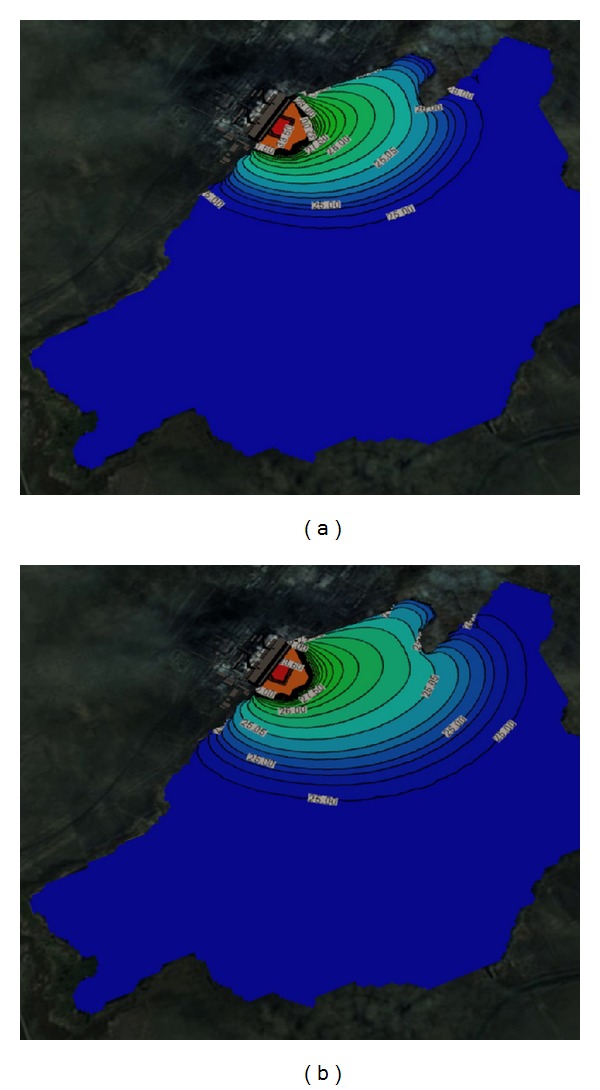
Outline and contours of temperature at 15 and 24 hours at the west wind after the launch of Ekibastuz SDPP-1 on the surface and the side view.

**Figure 4:**
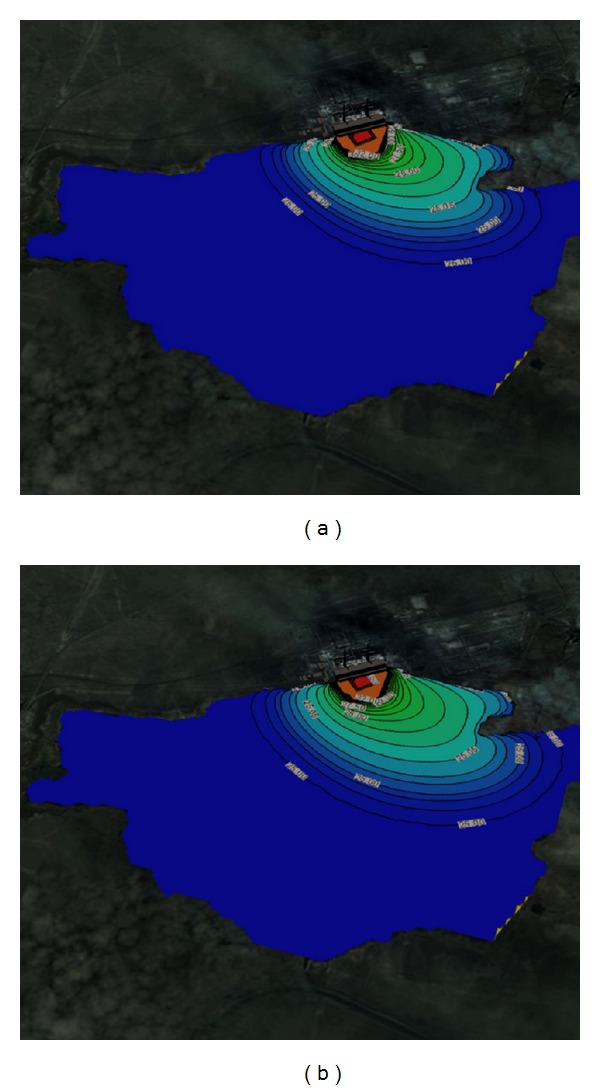
Outline and contours of temperature at 15 and 24 hours at the west wind after the launch of Ekibastuz SDPP-1 on the surface of the water and the top view.

**Figure 5:**
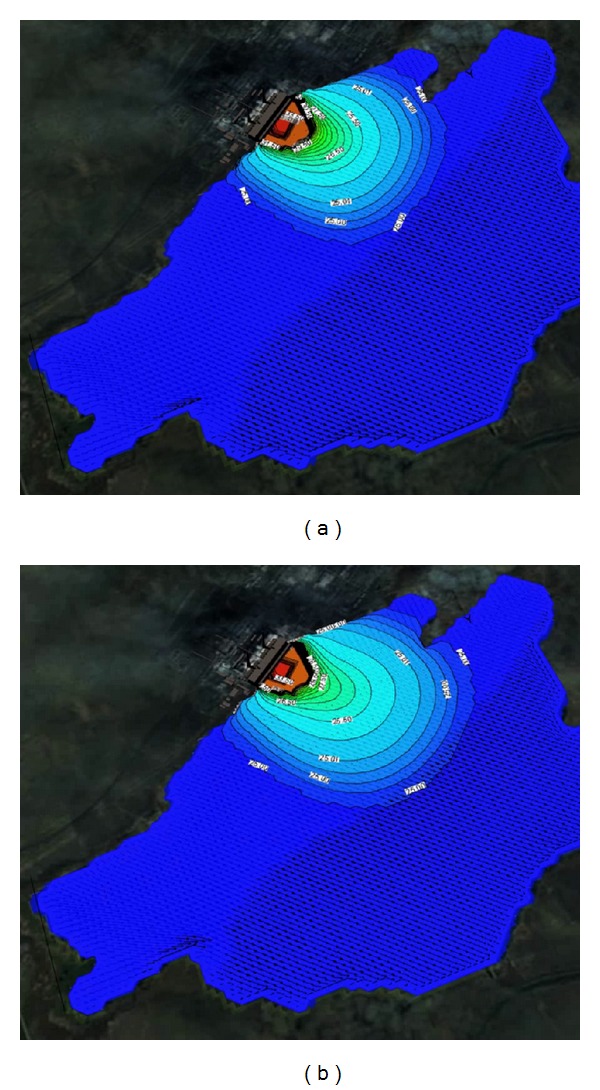
Outline and contours of temperature at 15 and 24 hours at the north-west wind after the launch of Ekibastuz SDPP-1 on the surface and the top view.

**Figure 6:**
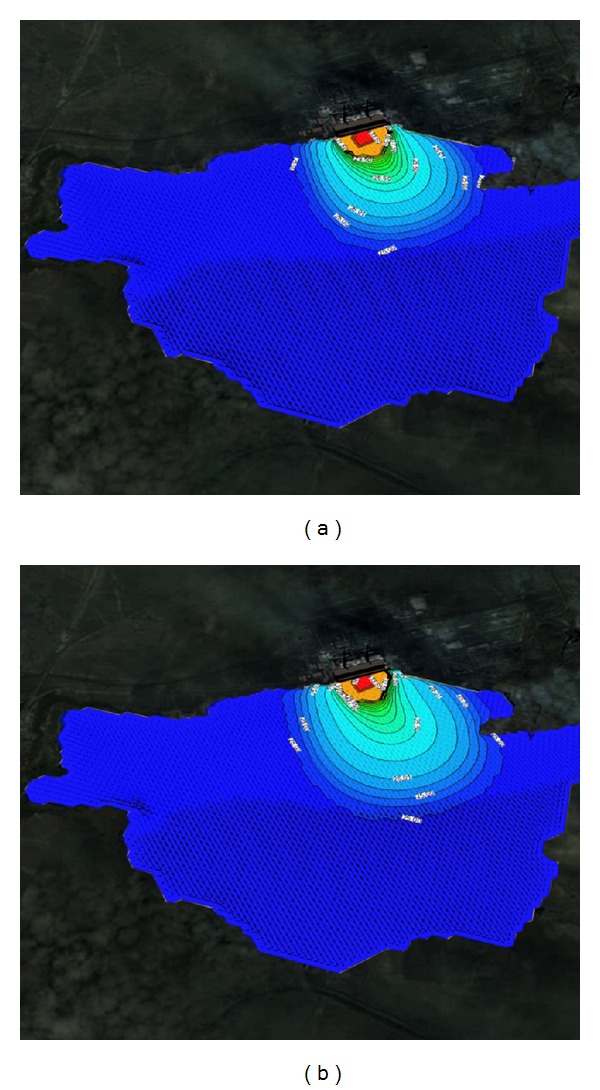
Outline and contours of temperature at 15 and 24 hours at the north-west wind after the launch of Ekibastuz SDPP-1 on the surface of the water and the side view.

Figures 1 and 2 show the solved three-dimensional spatial outline and contour of the temperature distribution at different times after the launch of Ekibastuz SDPP-1, on the surface, from different angles of view. Figures 3 and 4 show the solved spatial contour, contour of temperature at different times at the west wind after the launch of Ekibastuz SDPP-1, on the surface, from different angles of view. Figures 5 and 6 show the solved spatial contour, contour of temperature and velocity vectors at different times at the north-west wind after the launch of Ekibastuz SDPP-1, on the surface, from different angles of view. In all figures, we can see that temperature varies from 25°C to 33°C. Moreover, it can be observed that temperature on the surface of reservoir-cooler near Ekibastuz SDPP-1 is higher than that at a far distance from Ekibastuz SDPP-1. It means that mathematical model qualitatively describes the physical process. All the figures show that temperature distribution in some distance from Ekibastuz SDPP-1 approaches isothermal distribution. The results show that the temperature distribution is spread over the larger area of the reservoir-cooler. In all the figures, the simulation was done with different hydrometeorological conditions merged with real relief, which was taken from satellite pictures.

## 8. Discussions

LES is a more universal approach to close the system of equations which was filtered by Favre approach. A necessary condition for the performance of turbulent closures is “subgrid” model that correctly describes the dissipation of the kinetic energy of smoothed velocity fluctuations and the ability to simulate the circuit direct energy cascade from large to small eddies. This stage is the primary mechanism for the redistribution of energy in the inertial range of three-dimensional homogeneous isotropic turbulence. The principal advantage of the LES from RANS is that, due to the relative homogeneity and isotropy of the small-scale turbulence, plotting a subgrid model is much simpler than the used turbulence models for RANS, when it is necessary to model the full range of turbulence. For the same reason, the hope for a “universal” subgrid model for LES is much more reasonable than a similar model for RANS. These important benefits of LES increase significantly computational cost associated with the need (also for direct numerical simulation (DNS) case) of three-dimensional time-dependent calculations on sufficiently fine grids, even also in cases where direct interest in the practice of the average flow is two-dimensional and stationary. On the other hand, for obvious reasons, the computational resources which are required to implement the LES are much smaller than those for the DNS. The degree of influence of different processes governing the formation of stratified flows and hydrothermal conditions in the entire body of water can be divided into two zones. The first (near) zone is directly adjacent to the water of outlet structures. The second is for the major part of the reservoir. In the near zone, formation of the stratified flow is influenced by the processes of mixing discharged water with water from the reservoir. It should be regulated by creating a specific hydraulic regime in the outfalls. In the second, zone of hydrothermal regime is formed primarily by the processes of heat transfer. The propagation of heat in this part of the reservoir is more dependent on the wind (direction and speed). When you spread the heated water in a cold environment, density difference between the upper layer of warm water and bottom layer of cold water appears. This allows the use of a combined intake and discharge instead of building costly diversion canals to the discharge. Accordingly, this raises the problem of optimal choice of the geometrical and operational parameters of the reservoir-cooler for efficient work of power plant.

## 9. Conclusions

Thus, the usage of a mathematical model of three-dimensional stratified turbulent flow gives us a possibility which approximately qualitatively and quantitatively determines the basic laws of the hydrothermal processes occurring in the reservoir-cooler. Performed earlier from computational studies of hydrothermal regime of Ekibastuz SDPP-I reservoir-cooler, the velocity and temperature fields measurements were done. These fields have revealed the basic laws of hydrothermal and thermal fields in the water with stationary and under various hydrometeorological conditions. The distribution of temperature and passive scalar affect not only the processes of heat and mass but also the density stratification. Stratification appears in connection with the difference between the density of discharged water and the density of surrounding water in the pond, or the presence of impurities in the discharged water. For example, the heated water is light, so it is in the form of a jet or standing stretches near the free surface. Sustainable density stratification of water reduces turbulent exchange between the vertical layers of fluids, especially in the area with big difference. In general, hydrothermal regime of the reservoir is formed under the influence of uncontrollable natural factors (solar and atmospheric radiation, wind, convective heat transfer, evaporation, etc.) and the factors which may be adjusted (the amount and temperature of the discharged water, the presence of impurities, selective sampling, etc.).

More detailed mathematical model and data analysis are necessary to simulate more accurate thermal process in the reservoir-cooler. That is also relevant for future research direction too.

## References

[1] Lick W (1976). Numerical models of lakes currents. *Annual Review of Earth and Planetary Sciences*.

[2] Shang YP, Lick W, Gedsey RT, Molls FB (1978). Numerical computation of three-dimensional circulation in Lake Erie: a comparison of a free-surface model and a rigid-lid model. *Journal of Physical Oceanography*.

[3] Zhang YL, Baptista AM, Myers EP (2004). A cross-scale model for 3D baroclinic circulation in estuary-plume-shelf systems: I. Formulation and skill assessment. *Continental Shelf Research*.

[4] Oey LY (2006). An OGCM with movable land-sea boundaries. *Ocean Modelling*.

[5] Cheng RT, Casulli V Modeling a three-dimensional river plume over continental shelf using a 3D unstructured grid model.

[6] Fletcher CA (1988). *Computational Techniques for Fluid Dynamics: Specific Techniques for Different Flow Categories*.

[7] Roache PJ (1972). *Computational Fluid Dynamics*.

[8] Peyret R, Taylor TD (1983). *Computational Methods for Fluid Flow*.

[9] Tannehill JC, Anderson DA, Pletcher RH (1997). *Computational Fluid Mechanics and Heat Transfer*.

[10] Lowe SA, Schuepfer F, Dunning DJ (2009). Case study: three-dimensional hydrodynamic model of a power plant thermal discharge. *Journal of Hydraulic Engineering*.

[11] Issakhov A Mathematical modelling of the influence of thermal power plant to the aquatic environment by using parallel technologies.

[12] Issakhov A (2013). Mathematical modelling of the influence of thermal power plant on the aquatic environment with different meteorological condition by using parallel technologies. *Power, Control and Optimization*.

[13] Lesieur M, Metais O, Comte P (2005). *Large Eddy Simulation of Turbulence*.

[14] Tennekes H, Lumley JL (1972). *A First Course in Turbulence*.

[15] Tolstykh AI (1990). *Compact Difference Scheme and Their Applications to Fluid Dynamics Problems*.

[16] Issakhov A (2011). Large eddy simulation of turbulent mixing by using 3D decomposition method. *Journal of Physics*.

[17] Yanenko NN, Bunch JB, Rose DJ (1979). The method of fractional steps. *Space Matrix Computations*.

[18] Issakhov A (2012). Development of parallel algorithm for numerical solution of three-dimensional Poisson equation. *Journal of Communication and Computer*.

